# Multimodal single-cell analysis of nonrandom heteroplasmy distribution in human retinal mitochondrial disease

**DOI:** 10.1172/jci.insight.165937

**Published:** 2023-07-24

**Authors:** Nathaniel K. Mullin, Andrew P. Voigt, Miles J. Flamme-Wiese, Xiuying Liu, Megan J. Riker, Katayoun Varzavand, Edwin M. Stone, Budd A. Tucker, Robert F. Mullins

**Affiliations:** 1 University of Iowa Institute for Vision Research, Iowa City, Iowa, USA.; 2 Department of Ophthalmology and Visual Sciences and; 3 Medical Scientist Training Program, University of Iowa, Iowa City, Iowa, USA.

**Keywords:** Genetics, Ophthalmology, Genetic diseases, Mitochondria, Retinopathy

## Abstract

Variants within the high copy number mitochondrial genome (mtDNA) can disrupt organelle function and lead to severe multisystem disease. The wide range of manifestations observed in patients with mitochondrial disease results from varying fractions of abnormal mtDNA molecules in different cells and tissues, a phenomenon termed heteroplasmy. However, the landscape of heteroplasmy across cell types within tissues and its influence on phenotype expression in affected patients remains largely unexplored. Here, we identify nonrandom distribution of a pathogenic mtDNA variant across a complex tissue using single-cell RNA-Seq, mitochondrial single-cell ATAC sequencing, and multimodal single-cell sequencing. We profiled the transcriptome, chromatin accessibility state, and heteroplasmy in cells from the eyes of a patient with mitochondrial encephalopathy, lactic acidosis, and stroke-like episodes (MELAS) and from healthy control donors. Utilizing the retina as a model for complex multilineage tissues, we found that the proportion of the pathogenic m.3243A>G allele was neither evenly nor randomly distributed across diverse cell types. All neuroectoderm-derived neural cells exhibited a high percentage of the mutant variant. However, a subset of mesoderm-derived lineage, namely the vasculature of the choroid, was near homoplasmic for the WT allele. Gene expression and chromatin accessibility profiles of cell types with high and low proportions of m.3243A>G implicate mTOR signaling in the cellular response to heteroplasmy. We further found by multimodal single-cell sequencing of retinal pigment epithelial cells that a high proportion of the pathogenic mtDNA variant was associated with transcriptionally and morphologically abnormal cells. Together, these findings show the nonrandom nature of mitochondrial variant partitioning in human mitochondrial disease and underscore its implications for mitochondrial disease pathogenesis and treatment.

## Graphical abstract

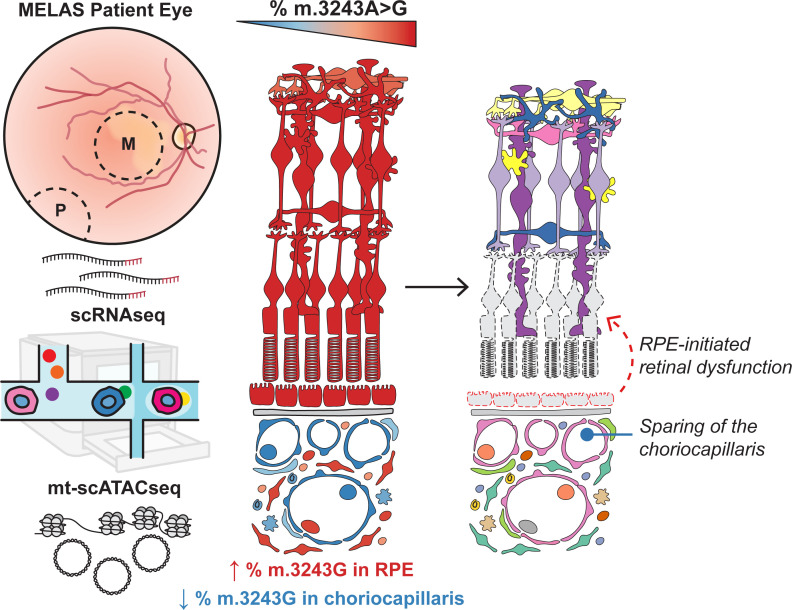

## Introduction

Several single-nucleotide variants on the mitochondrial genome are known to cause retinal dystrophy and multisystem disease (1). A notable feature of these variants is that they exhibit a high degree of phenotypic heterogeneity (2). This heterogeneity manifests in terms of timing of disease onset, organ systems involved, and severity of tissue dysfunction (3, 4). The heterogenous nature of genetic mitochondrial disease is likely due, at least in part, to the fact that the mitochondrial genome exists at a high copy number in relation to the nuclear genome (5). Since every cell possesses hundreds to thousands of mitochondrial genomes (mtDNA) (6), individual cells may contain a mixture of mutant and WT alleles, a phenomenon known as heteroplasmy (7). Mutant mitochondrial alleles are known to be unevenly distributed among organs (8, 9) and cells within the same organ (10, 11). How the distribution of mutant and WT mitochondrial molecules is established during organogenesis and maintained throughout life, as well as influence of this distribution on the resulting disease phenotype, are areas of active interest in the study of mitochondrial disease (3, 5, 12, 13).

Here, we focus on the most common pathogenic mitochondrial variant: m.3243A>G, a transition mutation found in the mitochondrial leucine tRNA gene *MT-TL1* (14). m.3243A>G is thought to disrupt electron transport chain function through mistranslation of the 13 protein-coding genes found on the mtDNA (15–20). Depending on the age of onset and organ system involvement, patients with this variant are typically diagnosed with 1 of 3 syndromic conditions: maternally inherited diabetes and deafness (MIDD, OMIM 520000); mitochondrial encephalopathy, lactic acidosis, and stroke-like episodes (MELAS; OMIM 540000); or Leigh syndrome (OMIM 256000) (20, 21). Symptoms can include vision loss (22–24), sensorineural hearing loss (25), diabetes mellitus, gastrointestinal disturbances, myopathy, stroke-like episodes (20), and premature death (26). Notably, the affected organs in these syndromes arise from all 3 primordial germ layers. In addition to causing dysfunction in a broad range of organ systems, the phenotypic expression of m.3243A>G is also highly variable, even within affected families (27). The heterogenous pattern of organ involvement between patients with the m.3243G variant has led to speculation that disease may be explained by the random partitioning of the pathogenic allele during early development, such that affected organs are simply those that randomly contain a higher proportion of m.3243G. However, emerging evidence has called some of the assumptions underpinning this model of m.3243A>G pathogenesis into question. Global mutant allele burden is not sufficient to explain the heterogeneity observed in m.3243A>G-associated disease phenotype (28, 29), implying a role for variable intratissue heteroplasmy. Recent work in circulating blood suggests that allelic segregation within tissues can be nonrandom (11). The concept of controlled mitochondrial DNA distribution builds on previous observations that mutant allele proportion followed predictable patterns between tissues (21, 30) and that such patterns may even be heritable, implying the involvement of nuclear genetic factors in governing the segregation or propagation of mutant alleles (3, 29).

In the current study, our goal was to investigate the relationship between the distribution of m.3243A>G across the retina and the clinical retinal phenotype in MELAS. We sought to test whether m.3243G was evenly distributed among the cell types of the human retina and to examine the effect of pathogenic variant dosing in specific ocular cell types on cellular phenotype. To accomplish this, we utilized primary postmortem human ocular tissue from patients diagnosed with m.3243A>G-associated MELAS. From this tissue, we collected neural retina, retinal pigment epithelium (RPE), and choroid (31). The retina, RPE, and choroid represent an ideal system for the study of the cell type–specific dynamics that govern heteroplasmy and cellular response to a mitochondrial variant in a complex tissue, as derivatives of neuroectoderm and mesoderm are in close physical and functional association. We provide evidence that the ratio m.3243G/m.3243A is not randomly distributed across the cell types of the neural retina and choroid. We identify dysregulation of transcriptional targets downstream of mTOR signaling that may act as mediators of heteroplasmy modulation in vivo. Understanding the process of heteroplasmy control by cells in vivo could enable heteroplasmy modulation as a therapeutic approach. Combining these intraorgan heteroplasmy data with prior knowledge of retinal metabolism and clinical manifestations of mitochondrial disease, we gain an increased understanding of m.3243A>G pathogenesis in the eye.

## Results

### Clinical ophthalmologic history of m.3243A>G disease.

Although m.3243A>G is known to be sufficient to cause disease (14, 32, 33), the sequence of events that connect the presence of the mtDNA mutation in cells to the widespread retinal atrophy is not fully understood (3, 29). Fundus examination of the proband revealed macular atrophy typical of the m.3243A>G variant (Supplemental Figure 1, A and B; supplemental material available online with this article; https://doi.org/10.1172/jci.insight.165937DS1) (22). Optical coherence tomography (OCT) of the retina showed outer retinal tubulations (ORTs) (Supplemental Figure 1C), a clinical finding known to be associated with m.3243A>G-associated disease and with RPE/choroid dysfunction, leading to retinal dystrophy more broadly (34–36). The pedigree structure was compatible with mitochondrial inheritance, and the proband had been previously confirmed to carry the m.3243A>G variant by sequencing and digital PCR (dPCR) (Supplemental Figure 3A). This patient was shown previously to harbor a relatively high global burden of the m.3243A>G variant in blood cells and skin fibroblasts compared with patients with less severe disease manifestations (30). Intratissue heteroplasmy distribution has not previously been explored in the eye.

### Single-cell profiling of MELAS retina and choroid.

**Figure 1:**
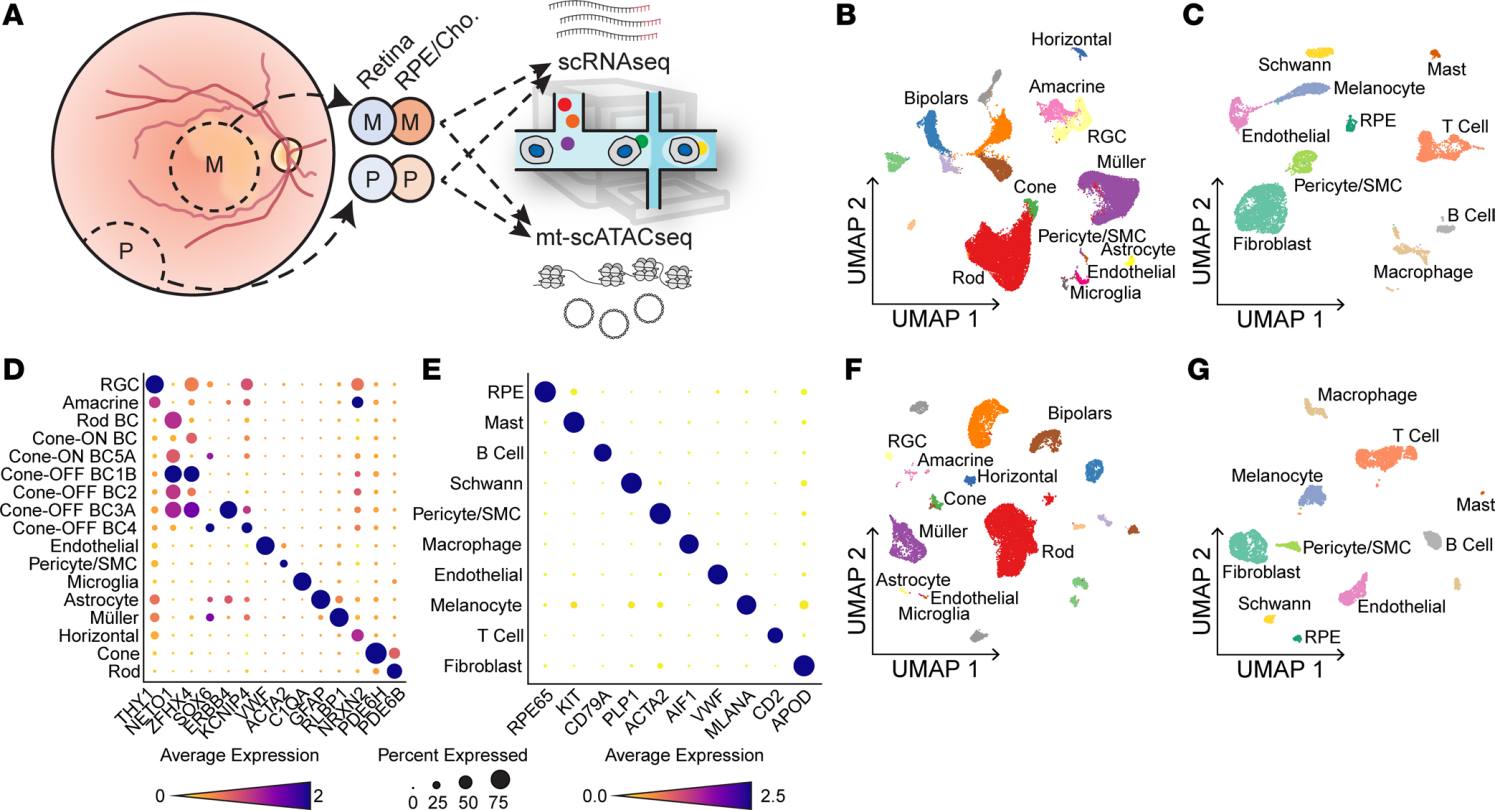
Transcriptome and chromatin accessibility profiling in single cells isolated from MELAS and control eyes. (**A**) Experimental schematic of scRNA-Seq and mt-scATAC–Seq studies. Macular (M) and peripheral (P) punches were dissected into retina and RPE/choroid samples. These samples were dissociated, split, and subjected to scRNA-Seq and mt-scATAC–Seq. (**B**) Two-dimensional UMAP embedding of neural retinal cells based on gene expression (scRNA-Seq) data from the MELAS proband and control donors. (**C**) Two-dimensional UMAP embedding of RPE, and choroidal cells based on scRNA-Seq data from the proband and control donors. (**D**) Dot plot indicating the magnitude of expression (color) and proportion of cells (size of dot) expressing known marker genes in each neural retinal cluster annotated in **B**. (**E**) Dot plot of curated marker genes in the each RPE/choroid cluster annotated in **C**. (**F**) Two-dimensional UMAP embedding of neural retinal cells based on chromatin accessibility (mt-scATAC–Seq) data from the proband and control donors. (**G**) Two-dimensional UMAP embedding of RPE, and choroidal cells based on mt-scATAC–Seq data from the proband and control donor. Cells with label transfer prediction score less than 0.6 are shaded in dark gray. RGC, retinal ganglion cell; SMC, smooth muscle cell; RPE, retinal pigment epithelium.

To better understand how the proportion of m.3243G varies within tissues and how such variability affects cellular dysfunction, we utilized a microfluidic-based single-cell sequencing approach to profile the transcriptome, nuclear chromatin accessibility, and heteroplasmy of single cells isolated from the neural retina and underlying RPE and choroid. Cells were isolated from the right eye (oculus dexter [OD]) of a patient with clinically and genetically diagnosed MELAS. Since m.3243A>G is known to cause region-specific retinal atrophy (22), samples were recovered from both the macular and peripheral retina as well as from that of an age- and sex-matched control individual. The neural retina and underlying RPE and choroid were each dissected, dissociated, and profiled with single-cell RNA-Seq (scRNA-Seq) and mitochondrial single-cell assay of transposase-accessible chromatin by sequencing (mt-scATAC–Seq) (37) (Figure 1A). Cells profiled by scRNA-Seq were integrated with data from 6 previously published scRNA-Seq samples of human retina and choroid (38, 39) (Table 1) and projected in a low-dimensional space based on gene expression (Figure 1, B and C); resulting clusters were manually annotated using previously described marker genes for human ocular cell types (39, 40) (Figure 1, D and E). Due to the sparseness inherent in the scATAC-Seq data, we annotated mt-scATAC–Seq clusters via unsupervised label transfer from the corresponding scRNA-Seq data sets (Figure 1, F and G) using previously described methods (41). An additional control sample of neural retina profiled by mt-scATAC–Seq was integrated with the 2 samples above. Clusters were examined to ensure proper integration of both control and proband samples and between cells taken from the macula or periphery (Supplemental Figure 3, E–H). The number of cells analyzed in each group after filtering for cell quality using either modality is shown in Table 2.

No major region-specific changes in gene expression were observed, and cells from these regions were computationally pooled for subsequent analyses. Since scRNA-Seq and mt-scATAC–Seq data sets were derived from the same initial samples, a high correlation between cell type proportion was observed (Supplemental Figure 3B), lending confidence to the unsupervised label-transfer approach. Confidence scores for predicted cell type classification were generated (Supplemental Figure 3, C and D), and only cells with a prediction score greater than 0.6 were used in downstream analysis. Cells not reaching this prediction score are colored in dark gray in Figure 1, F and G.

### Nonrandom m.3243G partitioning in the retina and choroid.

**Figure 2:**
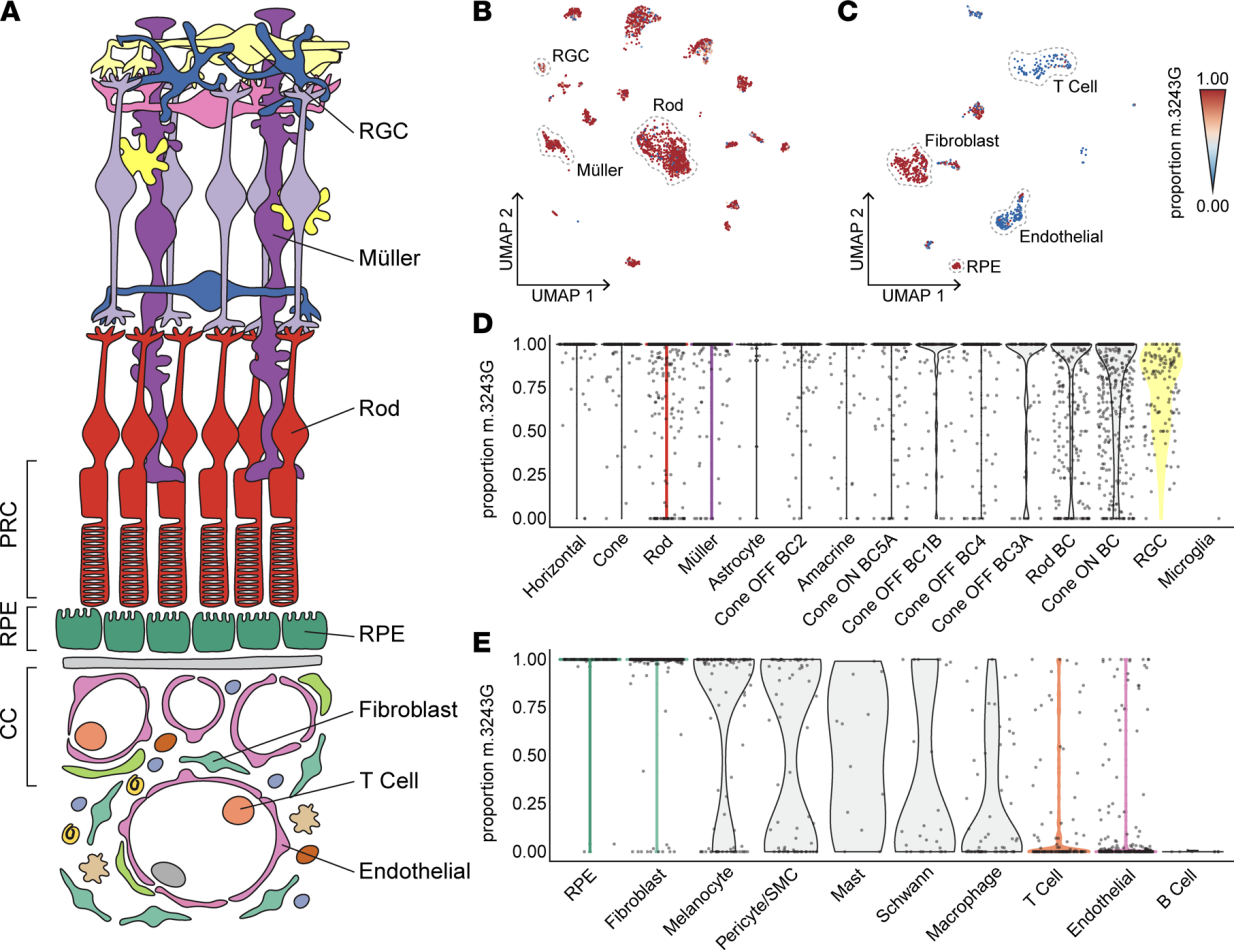
Nonrandom distribution of m.3243G in the retina and choroid measured by mt-scATAC–Seq. (**A**) Schematic of the neural retina, RPE, and choroid indicating the cell classes and regions captured by scRNA-Seq, mt-scATAC–Seq, and LCM-dPCR. (**B**) Two-dimensional UMAP embeddings of cells recovered from the MELAS neural retina. Individual cells are colored based on proportion of m.3243A>G mutant allele (red indicating 100% mutant allele and blue indicating 100% WT allele). (**C**) Two-dimensional UMAP embeddings of cells recovered from the MELAS RPE and choroid colored based on proportion of m.3243A>G mutant allele. (**D**) Violin plot demonstrating the proportion of m.3243A>G mutant allele in individual cells isolated from the neural retina sample as measured by mt-scATAC–Seq. Cells are grouped based on cell type cluster identity. (**E**) Violin plot demonstrating the proportion of m.3243A>G mutant allele in individual cells isolated from the RPE/choroid sample as measured by mt-scATAC–Seq. Cells included in **D** and **E** have at least 10***×*** coverage of the m.3243 locus.

Since the partitioning of the m.3243A>G variant within tissues would likely affect the resulting clinical phenotype, we asked whether heteroplasmy of the m.3243A>G variant differed among cells isolated from the neural retina and RPE/choroid (Figure 2A). mt-scATAC–Seq data were used, and only cells with sufficient coverage of the m.3243 locus were included in the analysis. To confirm that neither coverage of ATAC reads nor depth of mtDNA sequencing confounded heteroplasmy determination in single cells, these variables were plotted against m.3243G proportion for each cell included in Figure 2 and Supplemental Figure 4. No association between heteroplasmy calls and locus (Supplemental Figure 4, A and B) or mtDNA (Supplemental Figure 4, C and D) coverage was observed. The per-cell proportion of pathogenic m.3243G allele was overlayed onto cells projected in a low-dimensional space (Figure 2, B and C). Major cell types of the neural retina (photoreceptor cells, interneurons, and glia) were near homoplasmic for the pathogenic m.3243G allele (Figure 2B), all having median m.3243G proportions of 1. This finding was corroborated by bulk dPCR analysis of the neural retina (Supplemental Figure 3A). The RPE cells and fibroblasts of the RPE/choroid sample also had median m.3243G proportions of 1 (Figure 2C). However, endothelial and T cell populations were near homoplasmic for WT m.3243A, all having median m.3243G proportions beneath 0.05 (Figure 2C). The proportion of the pathogenic m.3243G allele within individual cells grouped by cell type is plotted in Figure 2, D and E. The melanocytes and pericytes from the RPE/choroid sample were heterogenous in terms of proportion of m.3243G (Figure 2E).

**Figure 3:**
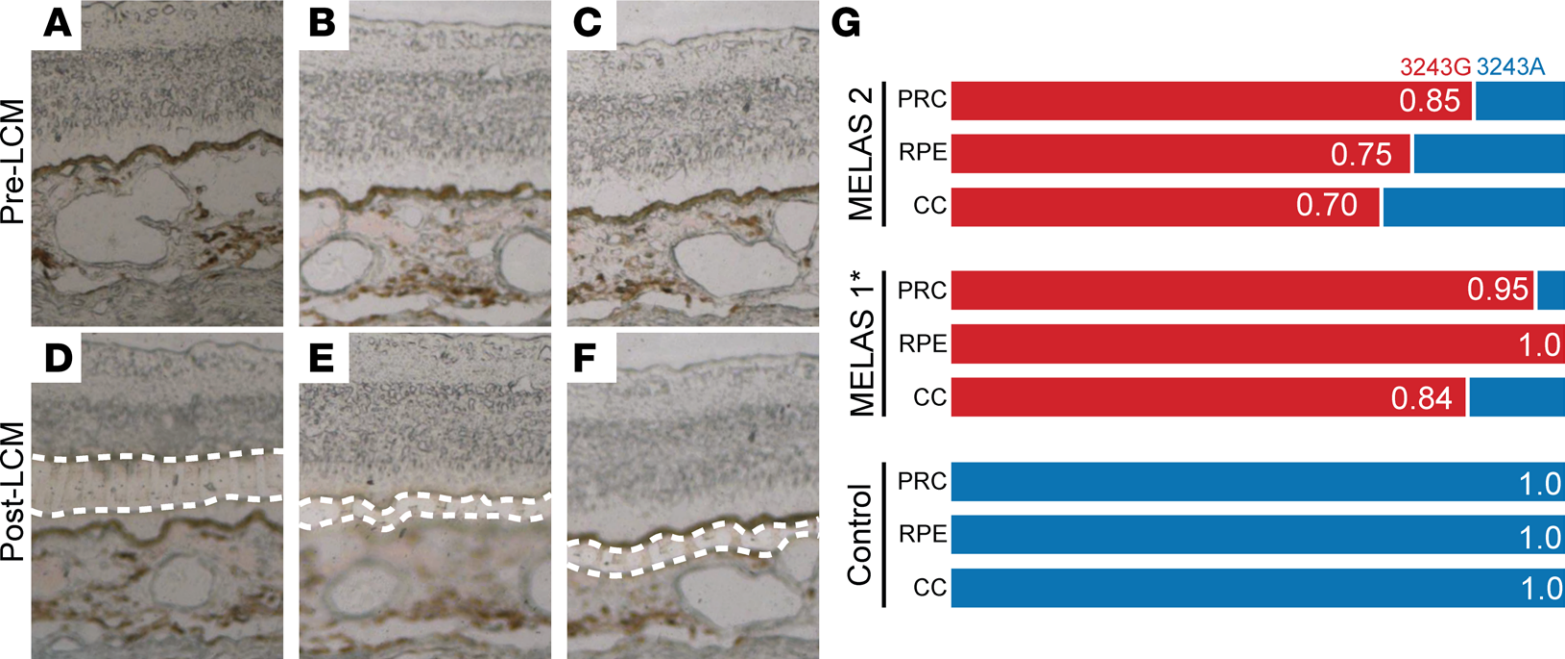
m.3243G partitioning in the retina and choroid measured by dPCR. (**A**–**C**) Color photomicrographs of neural retina (**A**), RPE (**B**), and choroidal (**C**) regions of fixed ocular sections. Regions of interest are shown before laser capture microdissection (Pre-LCM). (**D**–**F**) Color photomicrographs of fixed ocular sections after isolation of photoreceptors (PRC) (**D**), RPE (**E**), and choriocapillaris (CC) (**F**) by LCM (Post-LCM*)*. Regions isolated for subsequent dPCR are indicated with white dotted lines. (**G**) Proportion of mutant m.3243G in each LCM-captured region as measured by dPCR. MELAS 1* indicates proband eye contralateral to that used in the mt-scATAC study (Figure 2). MELAS 2 indicates an unrelated MELAS patient.

To validate the finding that choroidal endothelial cells have a reduced proportion of m.3243G compared with other choroidal and retinal cell types, we performed laser capture microdissection followed by dPCR (LCM-dPCR) on independent m.3243A>G ocular samples. Photoreceptor cells, RPE, and choriocapillaris (CC) layers (Figure 2A) were separately captured from the contralateral eye of the proband analyzed by mt-scATAC–Seq (MELAS 1*) as well as from an unrelated patient with MELAS known to carry the m.3243A>G variant (MELAS 2) (Figure 3, A–F). The CC showed routinely lower m.3243A>G than the RPE and photoreceptor cells layers (Figure 3G), consistent with the results of the mt-scATAC–Seq approach (Figure 2, B–E). Due to the highly complex and dense nature of the choroid, isolation of pure endothelial cells is not possible with LCM-dPCR, explaining the relatively elevated heteroplasmy compared with the mt-scATAC–Seq approach.

We used transmission electron microscopy to examine ultrastructural phenotype in retinal cells in MELAS. We found the mitochondria in MELAS neural retina (Supplemental Figure 2A) and RPE (Supplemental Figure 2C) to be swollen and hypertrophied compared with those in a control sample (Supplemental Figure 2, B and D).

### Dysregulated gene expression in retinal cell types with varying m.3243A>G proportion.

**Figure 4:**
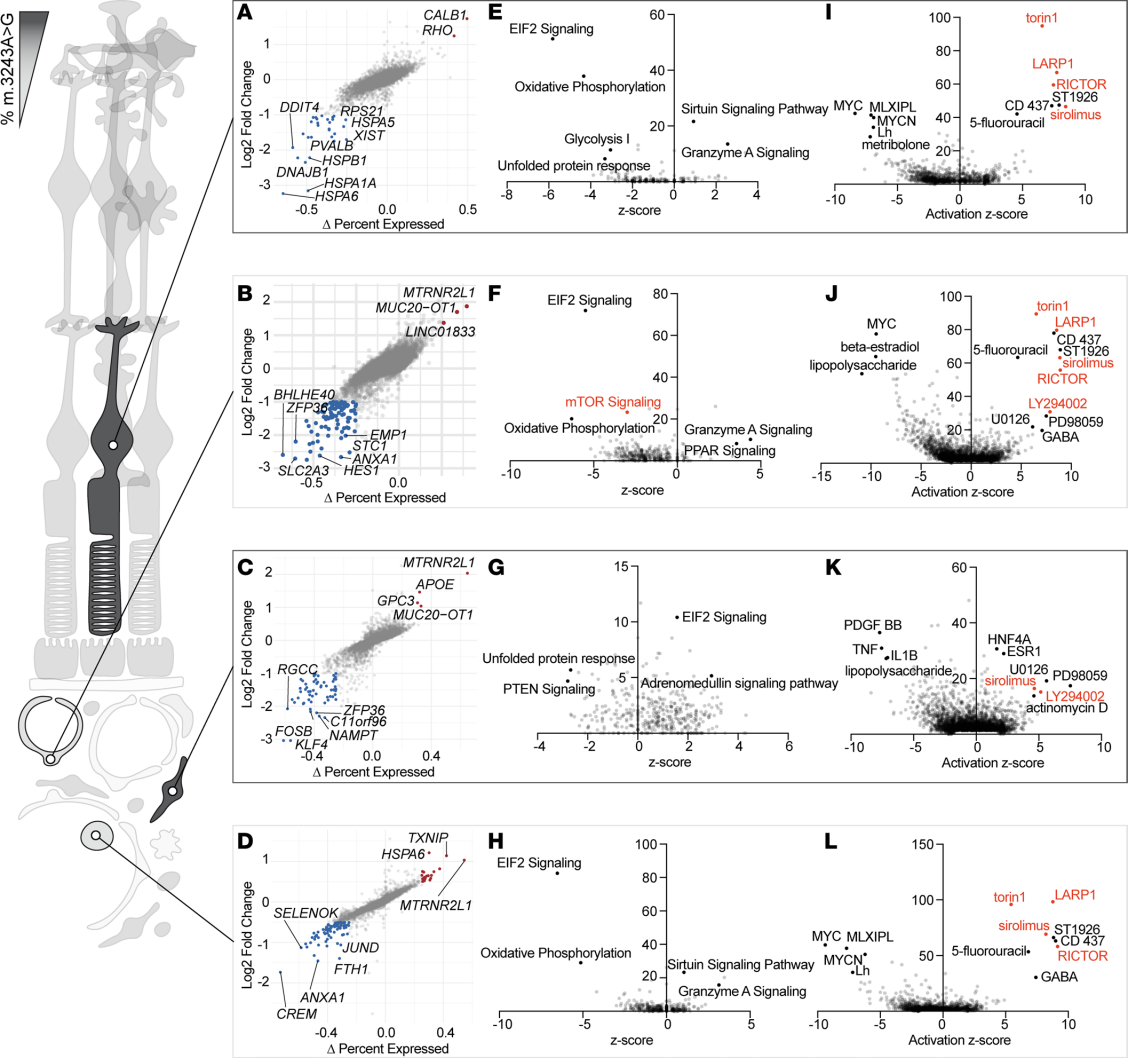
Impact of m.3243A>G heteroplasmy on gene expression of retinal cell types. (**A**–**D**) Differentially expressed genes between MELAS and control samples in cone photoreceptors (**A**), choroidal endothelial cells (**B**), choroidal fibroblasts (**C**), and T lymphocytes (**D**). Genes expressed with over 1.5-fold change between samples and Δ percent expressed greater than 25% are colored. The most highly differentially expressed genes are labeled. *MTRNR2L1* transcript is upregulated in MELAS samples in cell types with both high (**C**) and low (**B** and **D**) proportions of m.3243G. (**E**–**H**) Canonical pathways enriched in the dysregulated genes set are plotted. The gene expression profile associated with mTOR signaling is downregulated in the MELAS endothelial cell subset (**F**, red). (**I**–**L**) Predicted upstream regulators based on the cell type–specific gene list of dysregulated genes in MELAS versus control. Plotted molecules are experimentally shown to produce gene expression changes similar to those observed in differential expression analysis. The molecules torin1, LARP1, sirolimus, RICTOR, and LY294002 are known to act on the mTOR pathway are highlighted in red in **I**–**L**.

After determining the nonrandom partitioning of m.3243A>G across the cell types of the neural retina and RPE/choroid, we next asked how the presence of pathogenic m.3243G affects gene expression in different cell types. We performed differential expression analysis of cell types from the neural retina and RPE/choroid obtained from the MELAS proband and a normal control individual. We focused on 2 cell types with high levels of m.3243G (i.e., cone photoreceptor cells and choroidal fibroblasts) and 2 cell types with low levels of m.3243G (i.e., T lymphocytes and choroidal endothelial cells). We observed differential gene expression in both high and low m.3243G cell types (Figure 4, A–D). The nuclear encoded gene *MTRNR2L1* was upregulated in the 3 choroidal cell types in the MELAS samples compared with controls (Figure 4, B and C), irrespective of cell type–specific heteroplasmy. *MTRNR2L1* encodes the peptide humanin-like 1, a peptide that is highly homologous to humanin, which is encoded by the mitochondrial gene *MT-RNR2*. Humanin is known to be cytoprotective against oxidative stress and has been shown to rescue RPE cells from oxidative damage in vitro (42). *TXNIP,* the gene encoding thioredoxin interacting protein, was also upregulated significantly in MELAS endothelial cells and T cells compared with control (Figure 4, B and D). Upregulation of *TXNIP* was not observed in high-heteroplasmy cell types such as cone photoreceptor cells (Figure 4A). *TXNIP* expression has been associated with photoreceptor cells preservation in the context of retinal degradation through downregulating glucose utilization and shifting metabolism toward other fuel sources (43).

In order to understand the functional significance of dysregulated genes in the MELAS retina and choroid, we performed pathway analysis, looking for enrichment of canonical pathway terms (Figure 4, E–H) and for targets of known upstream regulatory factors or small molecules (Figure 4, I–L). Pathways involved in cellular energy production (Oxidative Phosphorylation, Glycolysis I), and the response to cellular stress (EIF2 Signaling) were differentially enriched in the control sample versus the MELAS sample in cell types with both high and low heteroplasmy (Figure 4, E–H). The mTOR signaling pathway was downregulated in low m.3243A>G–proportion endothelial cells (Figure 4F). Dysregulation of PI3K signaling, along with downstream AKT and mTOR signaling, has been shown previously in vitro to have a role in purifying selection against mitochondria carrying the m.3243A>G allele (44). Similarly, we found that the top upstream regulators that recapitulate the gene expression observed in MELAS cells involved inhibition of mTOR/PI3K/AKT signaling (Figure 4, I–L). These data indicate that the same pathway may play a role related to the control of pathogenic mtDNA alleles in the eye.

### Cell type–specific chromatin accessibility changes in MELAS.

**Figure 5:**
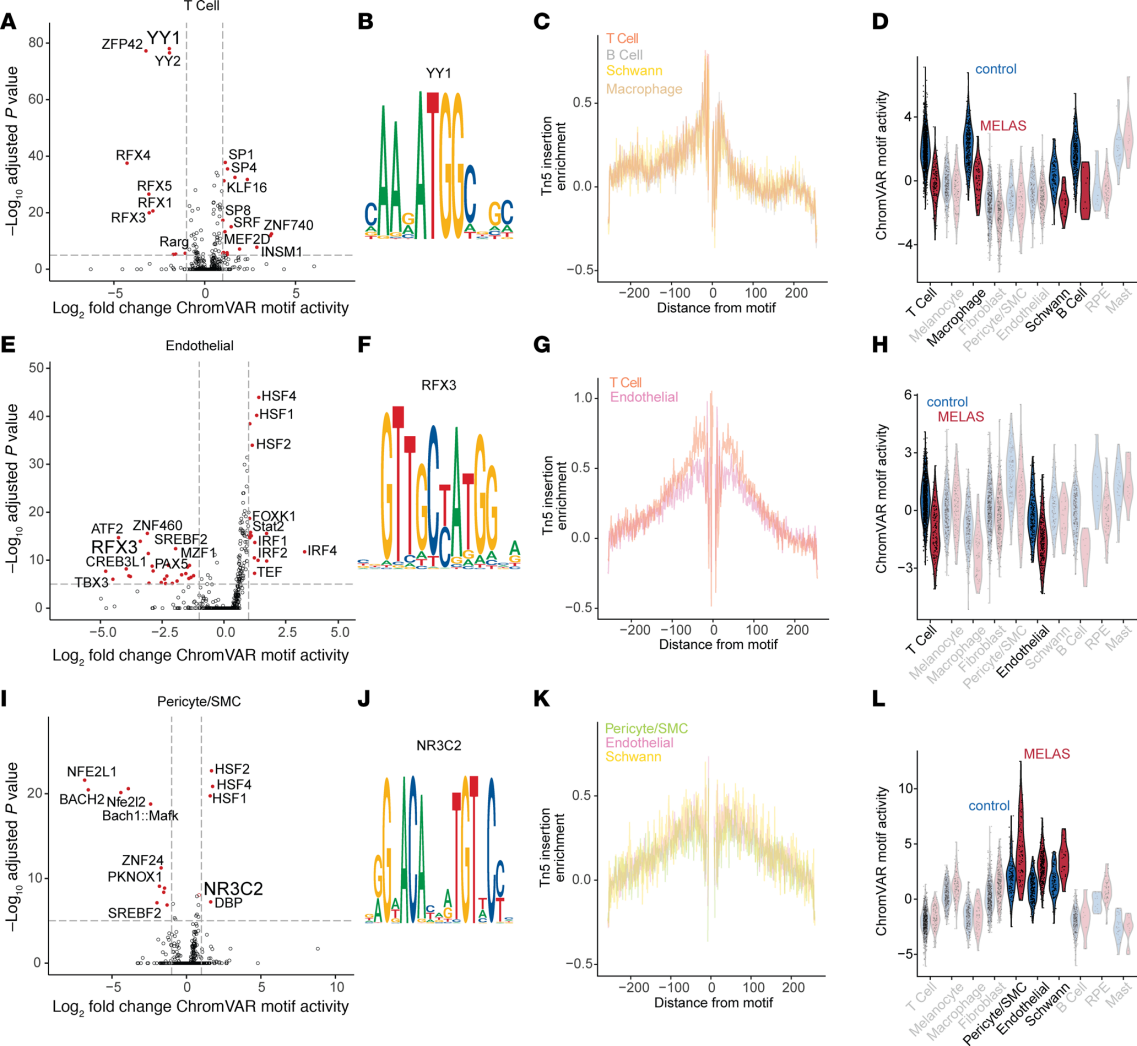
Differential accessibility of transcription factor binding motifs in MELAS choroid. (**A**) Top transcription factor binding motifs enriched in MELAS or control T cells. The YY1 and ZFP42 motifs are deenriched in MELAS T cells. (**B**) Motif logo of the JASPAR YY1 motif (MA0095.2). (**C**) Motif foot printing plot shows Tn5 transposase insertion enrichment in the locus flanking the YY1 binding motif. Traces for 4 cell types with lower YY1 motif enrichment in MELAS are shown. Foot printing traces represent accessibility in MELAS and control cells. (**D**) YY1 binding motif enrichment score shown for all choroidal cell types. Four cell types with low m.3243A>G heteroplasmy have lower YY1 binding motif enrichment in MELAS versus control. (**E**) Enriched motifs in choroidal endothelial cells. (**F**) RFX3 motif logo, one of several RFX factor motifs enriched in control endothelial cells versus controls. (**G**) RFX3 motif footprint plot showing traces for T cells and endothelial cells. (**H**) ChromVAR activity for the RFX3 motif is lower in MELAS endothelial and T cells in MELAS sample versus control. (**I**) Enriched motifs in the pericyte and SMC population of MELAS versus control. (**J**) The NR3C2 binding motif. (**K**) NR3C2 footprint plot indicating transcription factor-chromatin interaction in cell types of interest. (**L**) ChromVAR activity of NR3C2 is elevated in the low-heteroplasmy pericyte/SMC, endothelial, and Schwann cell populations in MELAS versus control.

Chromatin remodeling has previously been implicated in the cellular phenotype caused by m.3243A>G (45). We sought to use our mt-scATAC–Seq data to understand how the m.3243G variant alters transcription factor binding in retinal and choroidal cells. We used chromVAR (46) to identify transcription factor–binding motifs that were differentially enriched in MELAS versus control cells in specific choroidal cell types (Figure 5). Specifically, we performed differential enrichment analysis for MELAS versus control cells in the T cell (Figure 5A), endothelial cell (Figure 5E), and pericyte/SMC (Figure 5I) populations of the choroid. T cells and endothelial cells of the choroid exhibited near homoplasmy for the WT m.3243A allele, while the pericyte/SMC population displayed heteroplasmy for the m.3243A>G variant (Figure 2E).

We performed differential enrichment analysis of transcription factor binding motifs in MELAS versus control T cells and found YY1 to be a top hit. YY1 is a zinc-finger transcription factor known to work downstream of mTOR signaling to regulate mitochondrial function through transcription of nuclear-encoded mitochondrial genes (47). The YY1 binding motif (Figure 5B) and similar motifs showed decreased enrichment in T cells from the patient with MELAS as compared with the control (Figure 5A). These motifs were not differentially accessible in the homoplasmic endothelial population (Figure 5E). Footprint analysis of Tn5 insertion around the YY1 motif showed an expected patten of reduced accessibility at the motif, indicating blocking of transposase activity by bound YY1 (Figure 5C). YY1 motif enrichment was lower in MELAS than control in T cell, macrophage, Schwann cell, and B cell populations (Figure 5D), all of which had low proportions of pathogenic m.3243G allele (Figure 2E).

We next performed similar analyses on the endothelial (Figure 5, E–H) and pericyte/SMC (Figure 5, I–L) populations. We found that T cells and endothelial cells share reduced RFX3 motif enrichment in the MELAS sample as compared with control (Figure 5H). Pericyte/SMC, endothelial, and Schwann cell populations were all enriched for the NR3C2-binding motif in MELAS versus control cells (Figure 5, I–L). This motif was not enriched in T cells. NR3C2 activity has been previously linked to PI3K/ATK signaling (48), further supporting a connection between heteroplasmy modulation and signaling downstream of mTOR in the eye. Chemical inhibition of PI3K/AKT with LY294002 has been shown to reduce the proportion of m.3243G in vitro and to increase activity of NR3C2. Together, these analyses suggest that transcription factors acting downstream of mTOR signaling have differential activity depending on MELAS disease state and cell type.

### Multimodal sequencing reveals an aberrant gene expression profile in MELAS RPE.

**Figure 6:**
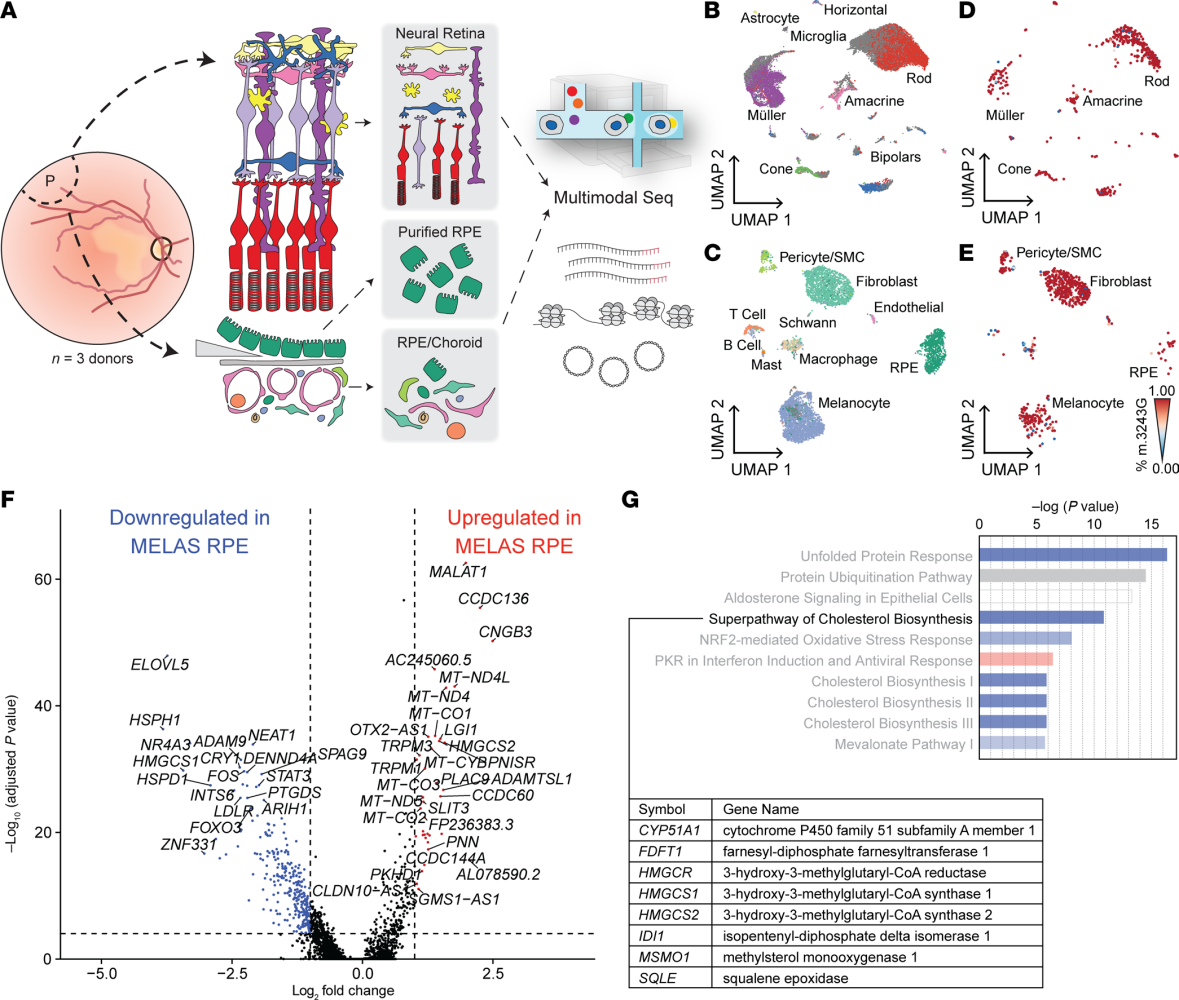
Multimodal sequencing of MELAS and control retina, RPE, and choroid cells. (**A**) Schematic of multimodal sequencing approach to measure gene expression, nuclear chromatin accessibility, and heteroplasmy in individual cells isolated from the retina and RPE/choroid. (**B**) Two-dimensional UMAP embeddings of neural retinal cells from the MELAS proband and controls based on weighted nearest neighbor (WNN) analysis of scRNA-Seq and mt-scATAC–Seq data. (**C**) Two-dimensional UMAP embeddings of RPE/choroidal cells from the MELAS proband and controls based on WNN analysis of scRNA-Seq and mt-scATAC–Seq data. (**D**) Two-dimensional UMAP embedding of neural retinal cells recovered from the proband. Individual cells are colored based on proportion of m.3243A>G mutant allele (red indicating 100% mutant allele and blue indicating 100% WT allele). (**E**) Two-dimensional UMAP embedding of RPE and choroid cells recovered from the proband samples. Individual cells are colored based on proportion of m.3243A>G mutant allele (red indicating 100% mutant allele and blue indicating 100% WT allele). (**F**) Volcano plot showing differentially expressed genes between the virtually homoplamic MELAS RPE and RPE cells from the control donors. (**G**) Pathway enrichment analysis for differentially expressed genes in **F**. Genes involved in cholesterol biosynthesis are deenriched in the MELAS RPE cells compared with control RPE.

RPE dysfunction has been implicated in the pathogenesis of m.3243A>G-associated vision loss (23, 49), and the ORTs frequently observed in this condition (Supplemental Figure 1C) are indicative of RPE cell death (50). Since the choroidal cells most responsible for photoreceptor cells health (endothelial cells) were homoplasmic for WT m.3243, we hypothesized that the RPE is the site of the initial tissue injury in the eye. To confirm the high proportion of mutant m.3243G in the RPE and investigating its impact on gene expression, we jointly profiled heteroplasmy, gene expression, and chromatin accessibility in single cells from neural retinal, choroidal, and purified RPE samples of the MELAS proband and controls (Figure 6A). Due to a low RPE cell count in unselected RPE/choroid samples (Figure 1, C and G), manually purified RPE samples were included in this experiment to guarantee sufficient cell number. Cell clustering was performed using weighted nearest neighbor (WNN) analysis (51), which combines gene expression and chromatin accessibility modalities in a weighted manner to drive and refine cluster identification (Figure 6, B and C) beyond that derived from single modalities (Supplemental Figure 5). The relative modality weighting behind each cluster is shown (Supplemental Figure 5, B and D). RPE and neural retina cells from the MELAS donor exhibited uniformly high burdens of m.324G3 (Figure 6, D and E).

To understand how the presence of the m.3243G allele affects RPE function in vivo, we performed differential gene expression analysis on MELAS and control RPE cells (Figure 6F). Several genes involved in lipid metabolism were differentially expressed (e.g., *ELOVL5, HMGCS1, LDLR, HMGCS2*). Pathway enrichment analysis showed downregulation of genes involved in cholesterol biosynthesis in the MELAS RPE as compared with control (Figure 6G). RPE cells are known to be a critical modulator of cholesterol homeostasis in the neural retina (52), indicating that a high proportion of m.3243G allele disrupts a key RPE function.

**Figure 7:**
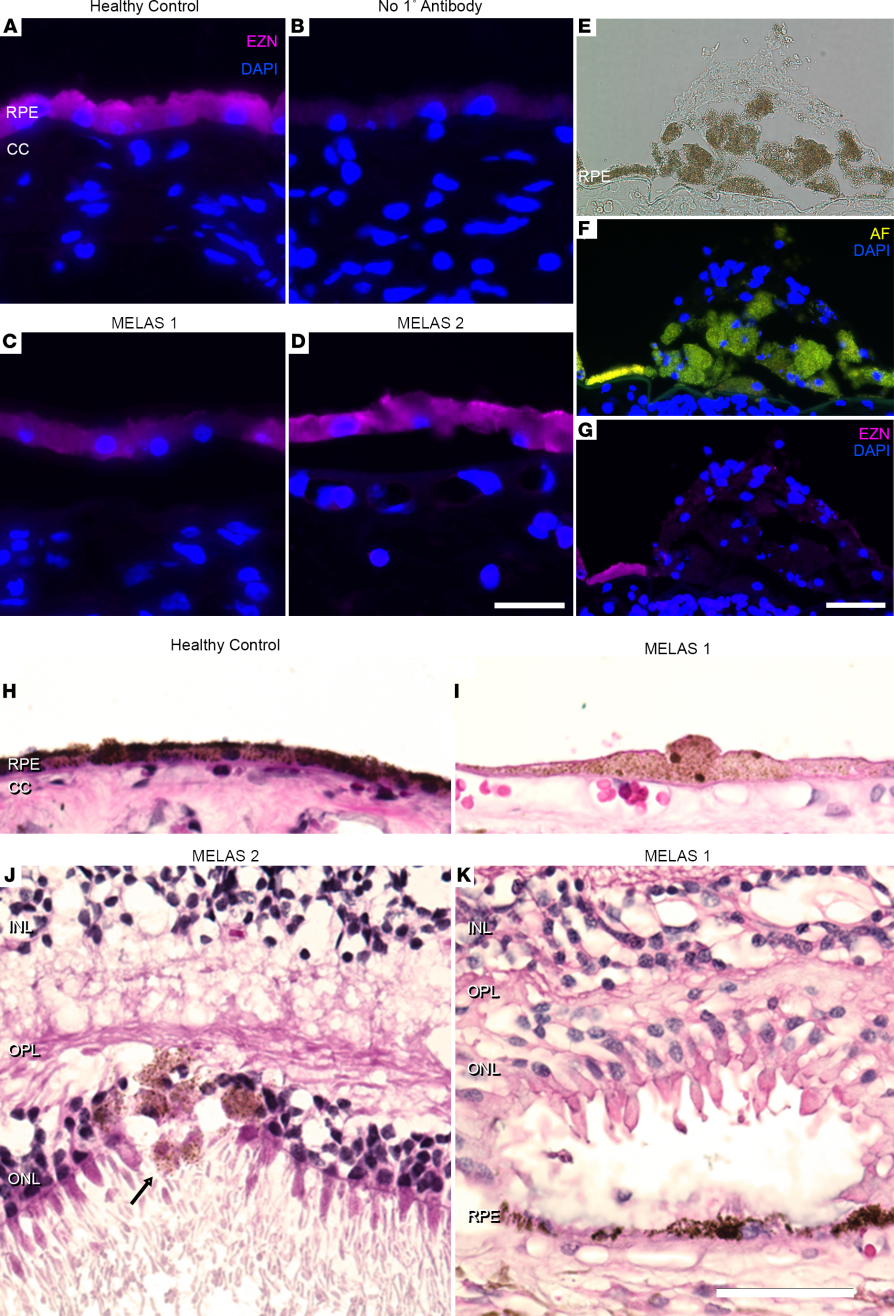
Abnormal RPE histology in MELAS patient eyes. (**A** and **B**) Ezrin staining in control RPE indicates proper polarization of mature epithelium. Panel **B** shows a negative control section stained with secondary antibody against mouse IgG but without primary antibody against EZR. (**C** and **D**) Dimmer and less polarized ezrin signal is observed in MELAS patient RPE. Scale bar: 25 μm (**A**–**D**). (**E**) A mass of pigmented cells at the layer of the RPE in a MELAS patient eye. (**F**) Pigmented cells display autofluorescence in the green and red channel (yellow overlay shown), indicating the presence of lipofuscin normally found in RPE cells. (**G**) Ezrin staining of the RPE layer is lost at the border of the pigmented lesion (Alexa Fluor 647 fluorescence pseudocolored magenta). Scale bar: 50 μm (**E**–**G**). (**H** and **I**) Compared with healthy donor RPE, MELAS RPE appears generally thinned and hypopigmented. Hypertrophied cells are observed. (**J**) Migration of pigmented cells into the neural retina is observed in a MELAS eye (arrow), indicating dedifferentiation of RPE. (**K**) Early-stage outer retinal tubulation over a patch of thinned RPE is observed in a MELAS eye. Scale bar: 50 μm (**E**–**G**). RPE, retinal pigment epithelium; CC, choriocapillaris; INL, inner nuclearlayer; OPL, outer plexiform layer; ONL, outer nuclear layer.

Functionally abnormal RPE cells in MELAS have been previously connected to loss of epithelial polarity and dedifferentiation of epithelial morphology (49). We next stained MELAS and control donor RPE for ezrin (EZR), a protein involved in cytoskeleton-membrane interaction and known to be a marker of epithelial polarity (53). MELAS RPE (Figure 7, C and D) exhibited decreased expression of EZR compared with the strong apical signal observed in control donors (Figure 7, A and B). Within the macula of a MELAS donor sample, we observed a multilayered clump of hypertrophic RPE cells (Figure 7E). Pigmented RPE cells outside the lesion stained for EZR, while those pigmented cells comprising the lesion did not (Figure 7G). Cells within the basal portion of the lesion exhibited autofluorescence, while apical cells did not (Figure 7F), consistent with epithelial-to-mesenchymal transition (EMT) of RPE in this MELAS sample. We performed H&E staining on 2 unrelated MELAS retinal samples to visualize gross RPE abnormalities. Both donor sections exhibited signs of RPE dysfunction and EMT that have been previously recognized in models of MELAS in vitro (49). MELAS donors exhibited RPE thinning (Figure 7, H and I), a recognized signed of RPE EMT (54), as well as cells with swollen cytoplasm. Additionally, migrating pigmented cells were visualized in the outer retina (55) (Figure 7J), and ORTs were observed (Figure 7K), presumably in response to underlying dysfunctional RPE. Together, these findings show that the m.3243G mutation in MELAS RPE is associated with transcriptionally and morphologically abnormal cells.

## Discussion

In this study, we sought to probe the distribution and consequences of m.3243A>G heteroplasmy in a clinically relevant human tissue. We profiled heteroplasmy, gene expression, and chromatin accessibility in single cells and found that m.3243G is nonrandomly distributed, being high in neural retina and RPE and lower in the choroidal endothelium. We also found that the presence of m.3243G resulted in dysregulation of genes involved in oxidative stress management and the mTOR/PI3K/AKT signaling pathway. Differential gene expression was observed in cell types lacking pathogenic allele, implying that this pathway may have a role in heteroplasmy modulation in vivo. We further show that transcription factor binding motif enrichment was dysregulated in MELAS in a cell type–specific manner and that factors involved in mTOR signaling were differentially active in the disease state. Finally, by combining previous ophthalmologic clinical observations (24, 56) and multimodal sequencing of the RPE, we show that the RPE dysfunction that is associated with high proportions of m.3243G was likely an early step in the pathogenic sequence that results in retinal dystrophy of MELAS and related mitochondrial conditions (i.e., MIDD and Leigh Syndrome).

### m.3243G is nonrandomly distributed between ocular cell types.

Mitochondria harboring pathogenic variants have long been known to be subject to purifying selection during oogenesis and early embryonic development (57). More recently, evidence has emerged that such selection may occur within certain tissues on a cell type–specific basis (10, 11, 58). Currently, the mechanism that leads to uneven mitochondrial DNA partitioning between cell types of the same individual is not clear. One hypothesis is that mitochondrial genomes are randomly partitioned into early embryonic progenitors during the first zygotic divisions. In such a model, high proportions of a mutant mtDNA in the CNS would arise from mutant mtDNA genomes being randomly partitioned into a neuroectoderm-fated cell in early embryonic development. However, reproducible patterns of mtDNA distribution across specific tissues of unrelated individuals (30) and within specific cell types within tissues of unrelated individuals (11) suggest that there is cell type–level control over mtDNA partitioning. Our data show that, within the microenvironment of the retina and choroid, certain cell types develop lower levels of m.3243G despite a background of globally elevated m.3243G in a severely affected patient. We found that choroidal T cells possessed low levels of m.3243G, building on previous observations of lower m.3243G in circulating T cells (11), and we observed a similar phenomenon in the endothelial cells of the choroid.

### The m.3243A>G variant causes macular retinopathy with evidence of RPE/choroid dysfunction.

It has been hypothesized that the unique macular specificity in m.3243A>G disease is caused by region-specific dysfunctional RPE cells (59). A notable anatomical feature of retinal dystrophy caused by m.3243A>G is the presence of ORTs (24, 34, 35), structures formed by cone photoreceptor cells reorganization driven by Müller cell activation (50). Clinical examination of the retina of patients with MELAS suggests a disease process that begins in the RPE and/or choroid and eventually leads to outer retinal damage. Our data showing a lack of the mutant allele in the choroidal endothelium support the hypothesis that the RPE is the key player in the formation of m.3243A>G-associated retinal disease.

The endothelial cells of the CC form in early development from a pool of hemanogioblasts that also give rise to primitive blood cells (60–62). The differentiation of mesenchymal progenitors that gives rise to the CC is distinct from the angiogenic process that later connects the choroidal circulation to the rest of the systemic circulation (60). Notably, the choroidal fibroblasts that displayed high m.3243G burden are also derived from the same mesenchymal progenitor pool, indicating that cell type–specific selection against the mutant allele as opposed to coincidental pattern following developmental lineages. The unique developmental origin of choroidal endothelium may explain the low proportion of m.3243G observed in this study as compared with a previous study of m.3243A>G in other endothelial cell populations (63).

### Retinal m.3243A>G heteroplasmy in the context of metabolism.

The metabolic program of retinal cells varies greatly by cell type (64, 65). Photoreceptor cells have the highest energy needs in the retina, primarily utilizing ATP to drive ion pumps that are essential for light modulated changes in membrane potential (65). However, photoreceptor cells generate most of their ATP through aerobic glycolysis, as opposed to the more efficient process of oxidative phosphorylation (66). While photoreceptor cells contain numerous mitochondria, their primary function may be to generate biosynthetic intermediates via the TCA cycle instead of ATP via the electron transport chain. Because of this, mistranslation of electron transport chain components in MELAS may negatively affect photoreceptor cells to a lesser degree than RPE.

Lactate generated by photoreceptor cell glycolysis is exported and utilized by glial and RPE cells as a substrate for oxidative phosphorylation in their mitochondria (64) in a reversed process reminiscent of the neuron/astrocyte metabolic linking elsewhere in the CNS. The reliance of the RPE on oxidative phosphorylation is necessary to support photoreceptor cell glycolysis, given their physical arrangement with respect to the choroid. Glucose arrives to the outer retina via choroidal blood flow and must be passed through the RPE layer to reach the photoreceptor cells (Figure 2A). Because of this arrangement, RPE preference for oxidative phosphorylation using lactate and other fuels allows for minimal glucose loss during transport to the photoreceptor cells under normal conditions (66). Additionally, RPE energy demand is supplemented by oxidation of ingested photoreceptor cell outer segment fatty acids (67).

Cell type–specific metabolic pathway preference appears to underlie the observed distribution of m.3243A>G burden across the retina and choroid and the pathogenic mechanism of the variant in the retina. The m.3243A>G variant is thought to impair tRNA charging by cognate aminotransferases (15, 68) and, as a result, likely disrupts electron transport chain protein translation (18). These changes, in turn, disrupt the process of oxidative phosphorylation in cells with high levels of m.3243A>G (20). It has been shown previously that cells harboring high levels of m.3243G are predisposed to favor glycolysis (44). Photoreceptor cells do not largely rely on the electron transport chain for energy production and may be more tolerant of the accumulation of mtDNA carrying the pathogenic allele. However, a high proportion of m.3243G in the RPE, as seen in the current study, likely causes decreased electron transport chain function and leads to derangement of the RPE-photoreceptor cells cooperative metabolic ecosystem, eventually causing photoreceptor cell dysfunction and vision loss.

### m.3243A>G-induced changes in transcription and chromatin accessibility involve mTOR signaling.

The nonrandom distribution of heteroplasmy observed in the eye invites the question of how heteroplasmy may be modulated in a cell type–specific manner. Understanding of such a phenomenon would open new avenues for treatment of mitochondrial disease. Previous work has shown that chemical inhibition of the mTOR/PI3K/AKT signaling axis is sufficient to reduce the proportion of the m.3243G allele in cultured cybrid cell lines (44). In our analysis of differential gene expression, we discovered that differentially expressed gene lists of cell types with both high and low m.3243G burdens were enriched for genes targeted by the same mTOR/PI3K/AKT inhibitors (i.e., sirolimus, LY294002, torin1) as well as endogenous modulators of the pathway (i.e., LARP1 and RICTOR). Motif enrichment analysis of mt-scATAC–Seq data further show dysregulation of transcriptional signaling downstream of mTOR in MELAS cells in low-heteroplasmy cell types in changes of YY1 and NR3C2 motif enrichment. Together, these analyses implicate mTOR signaling in the modulation of heteroplasmy in vivo, building on previous observations of its sufficiency to control heteroplasmy in vitro.

### Limitations of the current study.

The current study has limitations that necessitate future experimental work. Though m.3243A>G is the most common pathogenic mtDNA variant with an estimated individual frequency of 0.018% (69), cases of visual symptoms caused by this variant are still relatively rare, with an estimated 0.0002% of the population of the United States affected (70). As such, it is exceedingly difficult to acquire appropriate biological replicates of postmortem ocular tissues suitable for single-cell analysis. We validated our mt-scATAC–Seq findings of nonrandom heteroplasmy distribution using LCM-dPCR on independent biological samples (Figure 3). The data in the current study are further supported by previous observations of purifying selection against the m.3243G allele in circulating T cells. We observed the same trend in the choroidal T cells captured in this study, lending credence to our findings in other ocular cell types.

In this study, we focused only on the most common pathogenic mitochondrial variant. While our results imply a generalizable phenomenon for the establishment of mitochondrial variant segregation in the retina and choroid, further study is needed to clarify whether variants segregate in a generalized or variant-specific manner. The cell type specificity of pathogenesis in other retinal diseases caused by mtDNA variants such as Leber Hereditary Optic Neuropathy (LHON) and Neuropathy, Ataxia, and Retinitis Pigmentosa (NARP) provides evidence that some heteroplasmy modulation may be variant specific. Such knowledge may also clarify the underlying mechanism of mtDNA segregation within tissues.

### Conclusions.

In this study, we used a single-cell approach to measure m.3243G heteroplasmy in single cells of a developmentally complex tissue. We found that m.3243G proportion was lowest in the endothelial cells of the CC of 2 patients with MELAS and highest in the neural retina and RPE. We also found that, in cells with high m.3243G, chromatin accessibility and gene expression was altered. Together, these data support the hypothesis that high m.3243G in the RPE initiates MELAS-related retinal dystrophy. Observation of nonrandom m.3243G partitioning in adult samples implies selection against heteroplasmy in certain cell types during development. Ultimately, elucidation of how certain cells can regulate m.3243A>G heteroplasmy and other variants may lead to new therapeutic approaches for MELAS and other mitochondrial diseases.

## Methods

### Ocular tissue acquisition.

Human donor eyes used in single-cell studies from a patient affected with MELAS and an unaffected donor were acquired from the Iowa Lions Eye Bank. A second pair of eyes from a donor affected with MELAS was obtained from the Minnesota Lions Eye Bank. All eyes used in the study were obtained or preserved within 9 hours of death, and all experiments were performed in compliance with the Declaration of Helsinki and following full consent of the donor’s next of kin. Retinal and choroidal tissues were isolated from 8 mm trephine punch biopsies of the macular and inferotemporal peripheral regions and processed as previously described (40). Briefly, the neural retina was dissected away from the underlying RPE/choroid prior to tissue dissociation. Neural retina samples were dissociated with 20 units/mL of papain (Worthington Biochemical Corporation) with 0.005% DNase I (Worthington Biochemical Corporation) on a shaker at 37°C for 75 minutes. RPE/choroid samples were dissociated mechanically using razor blades into one-millimeter pieces then dissociated enzymatically using collagenase on a shaker at 37°C for 60 minutes. Following dissociation, cells were cryopreserved in DMSO-based Recovery Cell Cryopreservation Media (Thermo Fisher Scientific). After overnight cooling at –80°C, samples were transferred to liquid nitrogen (vapor phase) for long-term storage.

### Isolation of primary RPE.

Donor eyes were dissected as previously described (39). Following removal of the vitreous and neural retina, the RPE was manually scraped away from the underlying Bruch’s membrane and choroid. Cells were transferred directly to cryopreservation media and cryopreserved in the same manner as described for the dissociated neural retina and RPE/choroid above.

### scRNA-Seq library construction.

Cryopreserved cells were rapidly thawed at 37°C and resuspended in DPBS, no calcium, no magnesium (Thermo Fisher Scientific) with 0.04% nonacetylated BSA (New England Biolabs). Cells were filtered through a 70 μm filter and diluted to target 8,000 cells per run. Single cells were then partitioned and barcoded with the Chromium Controller instrument (10X Genomics) and Single Cell 3′ Reagent (v3.1 chemistry) kit (10X Genomics) according to the manufacturer’s specifications with no modification (Rev C). Final libraries were quantified using the Qubit dsDNA HS Assay Kit (Invitrogen) and diluted to 3 ng/μL in buffer EB (Qiagen). Library quality was confirmed using the Bioanalyzer High Sensitivity DNA Assay (Agilent) prior to sequencing.

### mt-scATAC–Seq library construction.

Cells were prepared for mt-scATAC–Seq using the protocol described previously by Lareau et al. (37). Briefly, cells were thawed as above and filtered through a 70 μm cell strainer. Cells were then washed twice with DPBS, no calcium, no magnesium (Thermo Fisher Scientific). Cells were fixed with 1% formaldehyde (MilliporeSigma) in PBS^–/–^ for 10 minutes at room temperature. Fixation was quenched with the addition of glycine (Research Products International) to a final concentration of 0.125M for 5 minutes at room temperature. Following fixation, cells were washed twice with ice-cold PBS^–/–^. Following the second wash, cells were resuspended in 100 μL ice-cold Lysis Buffer — 10 mM Tris-HCl (pH 7.4; MilliporeSigma), 10 mM NaCl (Invitrogen), 3 mM MgCl_2_ (Invitrogen), 0.1% Nonidet-P40 substitute (Research Products International), 1% Fraction V BSA (Research Products International) — and incubated on ice for 2.5 minutes. Following incubation, 1 mL of ice-cold Wash Buffer (10 mM Tris-HCl [pH 7.4], 10 mM NaCl, 3 mM MgCl_2_, 1% BSA) was added, and cells were pelleted at 500*g* for 5 minutes at 4°C. Cells were resuspended in 10 μL diluted Nuclei Buffer (10X Genomics) and counted using Trypan Blue (Thermo Fisher Scientific) with the Countess automated cell counter (Invitrogen). Based on these counts, cell suspensions were diluted in diluted Nuclei Buffer to a final target concentration of 6,000 cells per μL. Cells were then processed following the 10X Chromium Next GEM Single Cell ATAC Reagent Kit v1.1 User Guide (Revision D) without modification. Final libraries were quantified using the Qubit dsDNA HS Assay Kit (Invitrogen) and diluted to 3 ng/μL. Library quality was checked using the Bioanalyzer instrument (Agilent), wherein periodicity of fragment length was observed, consistent with high-quality ATAC-Seq library construction.

### Multimodal mt-scATAC and scRNA library preparation.

Cryopreserved RPE and choroid samples were thawed as described above. Cells were prepared for multimodal profiling using the sample preparation modifications described previously (71). Briefly, cells were thawed and filtered through a 70 μm cell strainer. Cells were then washed twice with DPBS, no calcium, no magnesium (Thermo Fisher Scientific). Cells were fixed with 1% formaldehyde (MilliporeSigma) in PBS^–/–^ for 10 minutes at room temperature. Fixation was quenched with the addition of glycine (Research Products International) to a final concentration of 0.125M for 5 minutes at room temperature. Following fixation, cells were washed twice with ice-cold PBS^–/–^. Following the second wash, cells were resuspended in 100 μL ice-cold Lysis Buffer — 10 mM Tris-HCl pH 7.4 (MilliporeSigma), 10 mM NaCl (Invitrogen), 3 mM MgCl_2_ (Invitrogen), 0.1% Nonidet-P40 substitute (Research Products International), 1% Fraction V BSA (Research Products International) — and incubated on ice for 2.5 minutes. Following incubation, 1 mL of ice-cold Wash Buffer (10 mM Tris-HCl pH 7.4, 10 mM NaCl, 3 mM MgCl_2_, 1% BSA) was added, and cells were pelleted at 500*g* for 5 minutes at 4°C. Cells were resuspended in 10 μL diluted Nuclei Buffer (10X Genomics) and counted using Trypan Blue (Thermo Fisher Scientific) with the Countess automated cell counter (Invitrogen). Cells were then processing following the 10X Genomics Chromium Next GEM Single Cell Multiome ATAC + Gene Expression (Revision E) without modification. Final libraries were quantified using the Qubit dsDNA HS Assay Kit and diluted to 3 ng/μL. Library quality was checked using the Bioanalyzer system.

### scRNA-Seq, preprocessing, and mapping.

scRNA libraries were pooled and sequenced using the NovaSeq 6000 instrument (Illumina) generating 100 bp paired end reads. Sequencing was performed by the Genomics Division of the Iowa Institute of Human Genetics. FASTQ files were generated from base calls with the bcl2fastq software (Illumina), and reads were mapped to the prebuilt GRCh38 reference with Cell Ranger v3.0.1 (10X Genomics) using the “count” function. Only cells passing the default Cell Ranger call were analyzed further. Neural retina and RPE/choroid samples were integrated separately using canonical correlation analysis (CCA) in Seurat v3.1 (41). Only cells with between 500 and 7,000 unique genes (features) were included in the analysis. For RPE/choroid samples, only cells with < 65% of reads mapping to mtDNA-encoded genes and < 40% of reading mapping to ribosomal RNA genes were included. For neural retina samples, only cells with < 40% of reads mapping to mtDNA-encoded genes and < 20% of reading mapping to ribosomal RNA genes were included.

### mt-scATAC–Seq, preprocessing, and mapping.

mt-ATAC libraries were pooled and sequenced using the Illumina NovaSeq 6000 instrument generating 100 bp paired end reads. Sequencing was carried out by the Genomics Division of the Iowa Institute of Human Genetics. FASTQ files were generated from base calls with the bcl2fastq software (Illumina). mt-scATAC–Seq reads were mapped to a modified GRCh38 reference genome that was generated by hard-masking nuclear regions that share homology with mitochondrial sequences, as described previously (37). Mapping and quantification were carried out by Cell Ranger ATAC (v2.0.0, 10X Genomics) using the “count” function, while the mt-ATAC portion of the multimodal library reads were mapped using Cell Ranger ARC (v.2.0, 10X Genomics). Only cells passing the default Cell Ranger call were analyzed further. Single-cell heteroplasmy of mitochondrial variants was determined using the mgatk package (v0.6.1).

Neural retina and RPE/choroid samples were integrated separately using Signac v1.4.0 (41). Briefly, integration anchors were identified with reciprocal LSI projection. LSI embeddings were then integrated using the IntegrateEmbeddings function, and a Uniform Manifold Approximation and Projection (UMAP) was generated. Only cells with peak region fragments between 1,000 and 75,000 and average mtDNA depth between 10***×*** and 1,000***×*** were used for integration and in downstream analyses. Manually annotated cluster labels were transferred from the scRNA-Seq data to the integrated mt-scATAC–Seq data. First, transfer anchors between the integrated scRNA-Seq and mt-scATAC–Seq objects were identified using the FindTransferAnchors function. Next, predicted cluster labels were generated using the TransferData function. Only cells annotated with a prediction.score > 0.6 were used in subsequent analysis.

### Multimodal sequencing, preprocessing, and mapping.

FASTQ files were generated from base calls with the bcl2fastq software (Illumina). Sequencing reads were mapped to a modified GRCh38 reference genome that was generated by hard-masking nuclear regions that share homology with mitochondrial sequences, as described previously (37). Mapping and quantification were carried out by Cell Ranger ARC (v2.0.0, 10X Genomics) using the “count” function.

Neural retina and RPE/choroid samples were integrated separately. For each set of samples, gene expression and ATAC libraries were integrated separately, and resulting embeddings were used to drive final clustering with WNN analysis.

For gene expression libraries, cells were filtered with the Seurat (v3.1) subset function. Neural retina cells with number of unique molecular identifiers (nUMIs) less than 100 (to remove cells with poor read quality) or greater than 4,000 (to remove cells likely to be doublets) were removed. RPE/choroid cells with nUMIs less than 500 or greater than 4,000 were removed. Neural retina cells with greater than 40% of reads originating from mitochondrial genes or 30% of reads originating from ribosomal genes were also removed. RPE choroid cells with greater than 30% of reads originating from mitochondrial genes or 20% of reads originating from ribosomal genes were also removed.

For each of the gene expression libraries, reads were normalized with the Seurat (v3.1) NormalizeData function, and 2,000 variable features were identified with the FindVariableFeatures command using the “vst” method. Integration anchors from the first 30 dimensions of the CCA were used to integrate gene expression data. Data scaling, principal component analysis, and clustering of the gene expression data were performed using Seurat (v3.1) (41).

For ATAC libraries, cells were filtered with the Signac (v1.4.0) subset function. Neural retina cells with fewer than 500 peak region fragments or greater than 15,000 peak region fragments were removed. RPE/choroid cells with fewer than 750 peak region fragments or greater than 15,000 peak region fragments were removed. Cells with greater than 1,000***×*** mtDNA depth were also removed. mtDNA sequencing depth (mtDNA_depth) was calculated using mgatk as described above. Term frequency inverse document frequency (TF-IDF) normalization and partial singular value decomposition were performed on each ATAC library using RunTFIDF() and RunSVD() (Signac) with default settings. Gene activities were computed using GeneActivity().

Integration anchors between ATAC data sets were identified using reciprocal LSI projection using FindIntegrationAnchors() in Signac. Dimensions 2–30 from the CCA were used. LSI embeddings were integrated using IntegrateEmbeddings based on these anchors and using dimensions 1–30.

Gene expression and ATAC portions of the experiment were combined using WNN analysis (Seurat). Integrated ATAC and gene expression objects were subset to include only common cells (i.e., cells that passed both ATAC and gene expression quality control thresholds). UMAP and cluster identification were performed on the resulting object. Following multimodal integration, cluster identities (i.e., cell type annotation) were identified using the gene expression portion of the experiment with the TransferData() function in Seurat from the annotated neural retina or RPE/choroid scRNA-Seq data obtained in this study (Figure 1, C and E). Transfer anchors between scRNA-Seq and multimodal datasets were identified using FindTransferAnchors with the following parameters: reduction = ‘pca’, dims = 1:30. Cells were annotated with a prediction score > 0.5.

### Single-cell heteroplasmy analysis.

Per-cell m.3243A>G heteroplasmy was determined as described by Walker et al. (11). mt-scATAC–Seq data were subset to retain only cells with coverage of the m.3243 locus greater than 10***×*** and less than 1.5 times the upper boundary of the interquartile range. Multimodal sequencing data were subset to retain only cells with coverage of the m.3243 locus greater than 5***×*** and less than the 99th percentile of cells. Only the proband (i.e., not control) patient was included in m.3243A>G heteroplasmy analysis. Of these subsets, m.3243A>G proportion was plotted in violin plots by cell type or as a color gradient on UMAP plots (Figure 2, B–E and Figure 6, D and E).

### Differential gene expression analysis.

Differential gene expression based on scRNA-Seq data was calculated between MELAS and control samples for each cell type annotated. The FindMarkers() function (Seurat v4.0.3) was used with default parameters. The difference in the percent of cells expressing a given gene (Δ Percent Expressed) was calculated for each gene in each cell type. Genes labeled in Figure 4 had a log(fold change) ≥ 1 and Δ Percent Expressed ≥ 0.25. Differential gene expression based on the scRNA-Seq portion of the multimodal sequencing data was performed using the FindMarkers() function (Seurat v4.0.3) with default parameters. Genes with adjusted P < 1 ***×*** 10^–5^ and fold change > 2 were considered as significantly differentially expressed in Figure 6.

### Pathway enrichment analysis.

All genes with an adjusted *P* ≤ 0.05 from the above differential expression analysis were used for pathway enrichment analysis with Ingenuity Pathway Analysis (version 01-21-03, Qiagen). Enriched canonical pathways were identified, and upstream analysis was performed to identify regulators known to activate gene expression patterns similar to those observed in enriched genes. All regulators with an activation *Z* score ≥ 2 were plotted in Figure 4.

### Transcription factor binding motif enrichment analysis.

Transcription factor binding motif enrichment (ChromVAR Motif Activity) was computed on a per-cell basis using ChromVAR run in Signac (46). The JASPAR 2022 database of motif position frequencies was used (72). Differential motif enrichment analysis was performed using the FindMarkers function of Seurat (4.0.5), using a logistic regression model and likelihood ratio test (test.use = LR) with the number of ATAC counts as latent variables (latent.vars = nCount_ATAC). Motif enrichment volcano plots were generated using EnhancedVolcano (1.8.0). Motif footprint analysis was carried out using the Footprint function of Seurat with the UCSC hg38 reference genome and default parameters.

### Transmission electron microscopy.

Donor samples were fixed in half-strength Karnovsky’s fixative and processed for TEM as described previously (34). Sections were cut at 85 nm with an ultramicrotome (Leica) and collected on copper slot grids coated with Formvar (0.5%); images were collected on a Hitachi HT7800 transmission electron microscope.

### Immunofluorescence staining of human donor RPE.

Cryostat sections of 2 MELAS and 5 control formaldehyde-fixed human donor maculae were collected. Sections were blocked in 1 mg/mL BSA for 15 minutes and labeled with antibodies directed against the *EZR* gene product Ezrin (Invitrogen, MA5-13862, clone 3C12) at a 1:50 dilution. Following 1 hour of incubation, slides were rinsed in PBS (with 1 mg/mL MgCl_2_ and CaCl_2_) and incubated with Alexa Fluor 647–conjugated (a fluorophore with excitation/emission maxima outside of those of RPE lipofuscin; Figure 7B) donkey anti–mouse IgG (Invitrogen, A31571, 1:200). Slides were counterstained with DAPI and imaged on a fluorescence microscope. Representative images are shown.

### LCM.

Fixed frozen blocks containing neural retina and choroid from both MELAS donors and an unaffected control donor were sectioned at 7 μm thickness onto membrane slides (Zeiss). Prior to sectioning, slides were exposed to UV light for 30 minutes in a tissue culture hood, per manufacturer’s suggestion. Mounted sections were rinsed once with cold deionized water prior to LCM. LCM was carried out using the PALM Combi LCM system (Zeiss). Regions of interest (i.e., photoreceptor cells, RPE, or CC) were traced and captured into adhesive cap tubes following the manufacturer’s recommended protocol. DNA was isolated using the Arcturus PicoPure DNA Extraction Kit (Applied Biosystems). Briefly, samples were digested overnight in a Proteinase K solution at 65°C, followed by a 10-minute inactivation at 95°C. DNA was stored undiluted at –20°C.

### Genotyping by dPCR.

Custom probe-based assays were designed against either variant at the m.3243 locus as previously described (30). Probes were designed incorporating several locked nucleic acids (indicated with “+” below) to attain adequate binding specificity to provide allelic specificity (Table 3) and manufactured by Integrated DNA Technologies. dPCR was carried out using the 3D PCR platform (Applied Biosystems). For the m.3243A/G assay, the following mixture was used (per reaction): 8.7 μL 3D Master Mix V2 (Applied Biosystems), 0.85 μL A-FAM Probe (5 μM), 0.85 μL G-HEX Probe (5 μM), 1.67 μL F Primer (5 μM), 1.67 μL R Primer (5 μM), 1.66 μL H_2_O, and 1 μL DNA lysate from LCM. In total, 14.5μL of the mixture was loaded into a 3D Digital PCR chip using the QuantStudio Digital PCR Chip Loader following the manufacturer’s instruction.

The thermal cycler program was run as follows for the m.3243A/G assay. Step 1: 95°C, 10 minutes; Step 2: 56°C, 2 minutes; Step 3: 98°C, 30 seconds; and Step 4: 60°C, 2 minutes. Steps 2 and 3 were cycled 45 times. After amplification, chips were allowed to equilibrate to room temperature for approximately 10 minutes and were then read. Data were analyzed using the 3D Quantstudio cloud-based web application, and the automatically generated thresholds were used. The proportion of G allele for the *MT-TL1* A/G heteroplasmy assay was calculated as: 

  
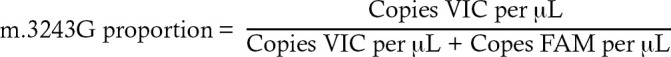
Equation 1

### Human donor samples.

Human donor sample characteristics are summarized in Table 1. Reprocessed human donor scRNA-Seq data were accessed through the NCBI Gene Expression Omnibus (GEO) under accession nos. GSE169047 and GSE135922.

### Data availability.

All raw and processed sequencing data generated in this study have been submitted to the NCBI GEO (https://www.ncbi.nlm.nih.gov/geo/) under accession no. GSE202747. Code used for sequencing analysis and figure generation is available at the following GitHub repository: https://github.com/nkmullin/melas_jci_insight_2023 (commitID f65471b).

### Statistics.

Differentially expressed genes between disease conditions were identified using a Wilcoxon Rank Sum test. The *P* value was adjusted based on Bonferroni correction using all genes in the data set. Differential motif enrichment analysis was performed using a logistic regression model and a likelihood ratio test with the number of ATAC counts used as latent variables.

### Study approval.

Human donor eyes used in this study were acquired from the Iowa Lions Eye Bank or the Minnesota Lions Eye Bank in accordance with the Declaration of Helsinki following consent of the donor’s next of kin. The human participant aspects of the study were approved by the IRB of the University of Iowa. The patients for whom clinical data are available were seen at the University of Iowa Hospitals and Clinics and provided written informed consent for inclusion in this study.

## Author contributions

NKM, APV, BAT, and RFM designed the study. NKM, APV, MJFW, XL, MJR, and KV conducted experiments and acquired data. EMS examined the proband and provided clinical data. NKM and APV analyzed the data. NKM wrote the original draft of the manuscript. APV, EMS, BAT, and RFM edited the manuscript. All authors reviewed and approved the final manuscript.

## Supplementary Material

Supplemental data

## Figures and Tables

**Table 3:**
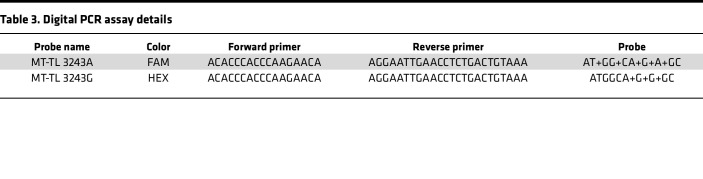
Digital PCR assay details

**Table 1:**
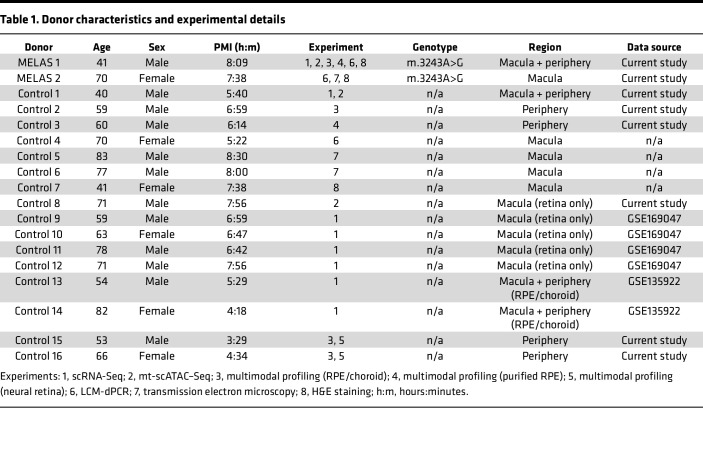
Donor characteristics and experimental details

**Table 2:**
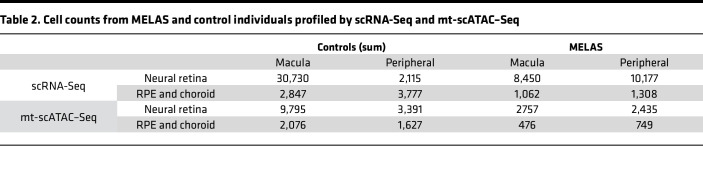
Cell counts from MELAS and control individuals profiled by scRNA-Seq and mt-scATAC–Seq

## Version 1 06/08/2023

In-Press Preview

## Version 2 07/24/2023

Electronic publication
