# Supplementary material for: Multimodal single-cell analysis of nonrandom heteroplasmy distribution in human retinal mitochondrial disease
Source: JCI Insight. 2023 Jul 24;8(14):e165937. doi: 10.1172/jci.insight.165937 (PMC10443808; doi:10.1172/jci.insight.165937)
Supplement: Supplemental data [file jciinsight-8-165937-s014.pdf]

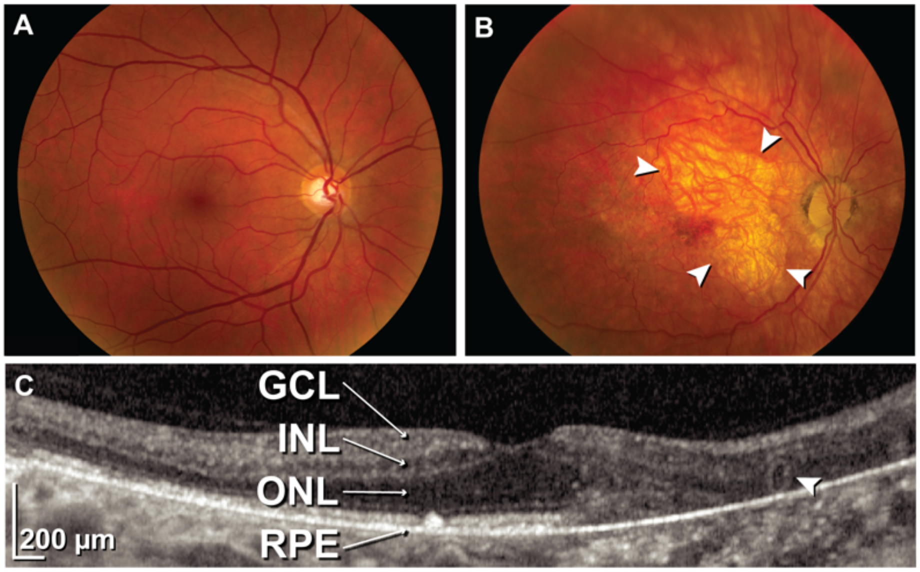

Supplemental Figure 1. Macular retinopathy caused by the m.3243A>G variant. (A) Color fundus photograph of the right eye of a healthy control patient with 20/20 acuity. **(B)** Color fundus photograph of the proband. Macular atrophy of the RPE and choriocapillaris typical of MELAS is appreciable (white arrowheads). **(C)** Optical coherence tomography (OCT) of the proband demonstrates outer retinal tubulations (white arrowhead) indicative of migration of cone photoreceptor cells following RPE cell death (GCL; ganglion cell layer, INL; inner nuclear layer, ONL; outer nuclear layer, RPE; retinal pigment epithelium/Bruch's complex).

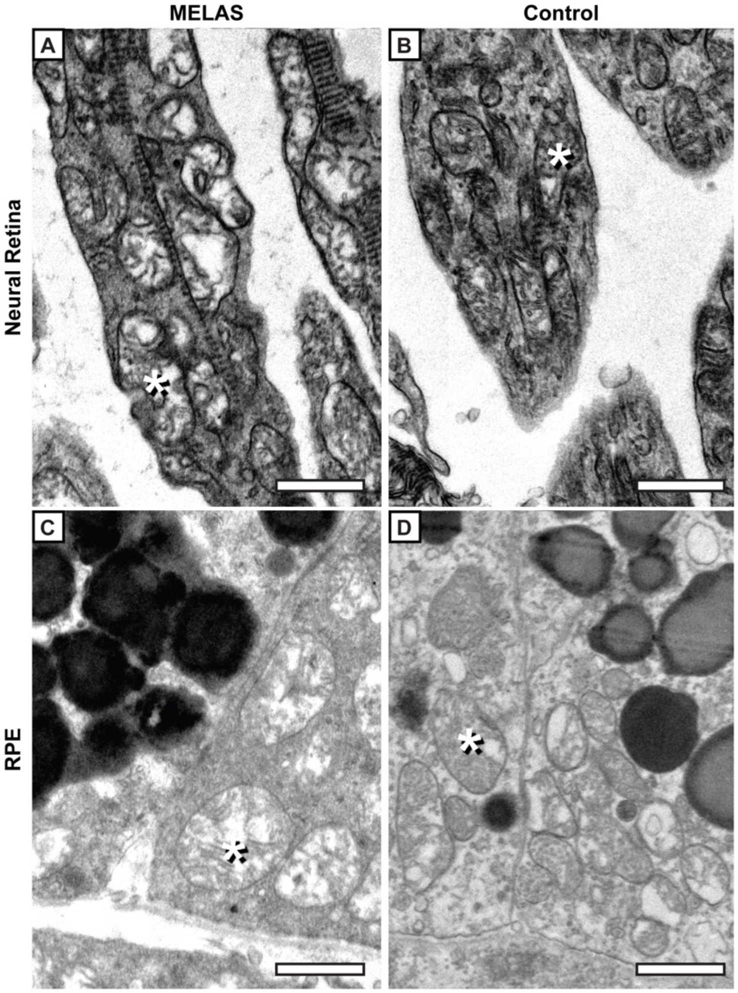

Supplemental Figure 2. Mitochondria ultrastructure in MELAS neural retina and RPE. (A) Transmission electron micrographs of photoreceptor cell inner segments from MELAS patient samples reveal abnormal mitochondrial structure compared to control tissue **(B)**. **(C)** Transmission electron micrograph of RPE cells from MELAS patient samples also show abnormal mitochondrial structure compared to control (**D**). Mitochondria are indicated with asterisks. Scale bar represents 1μm.

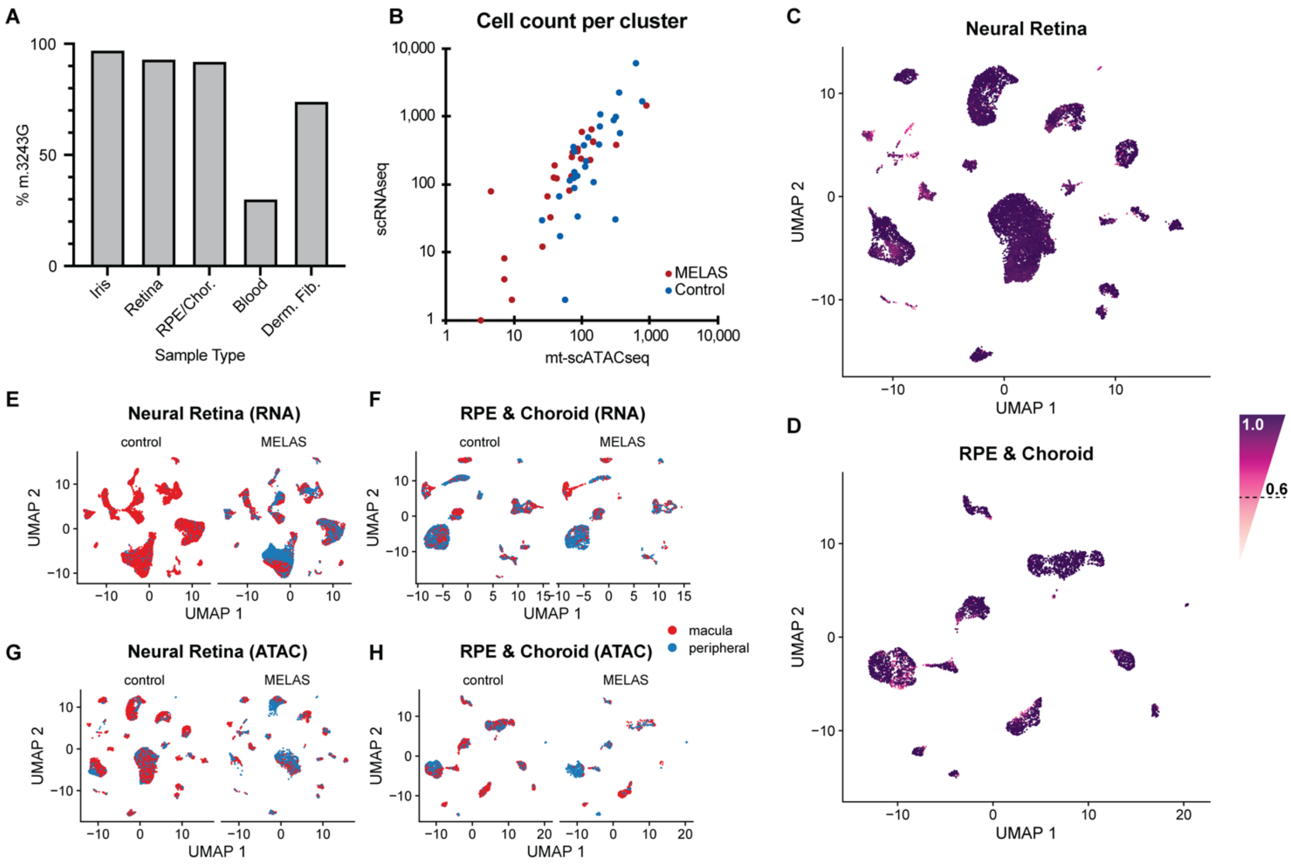

Supplemental Figure 3. scRNAseq and mt-scATACseq of MELAS and control ocular cells. (A) m.3243A>G heteroplasmy as measured by digital PCR in ocular and non-ocular cell types from the proband (MELAS 1). **(B)** High correlation in cell number per cluster as measured by scRNAseq and mt-scATACseq modalities across MELAS and control samples. Each dot represents one cell type in the conrol (blue) or MELAS (red) donor. **(C)** Two-dimensional UMAP embedding of neural retinal cells based on mt-scATACseq data from the proband and control donor. Cells are colored based on label transfer prediction score. **(D)** Two-dimensional UMAP embedding of RPE and choroidal cells based on mt-scATACseq data from the proband and control donor. Cells are colored based on label transfer prediction score. **(E, F)** Two-dimensional UMAP embedding of neural retinal and RPE/choroidal cells based on scRNAseq data. **(G, H)** Two-dimensional UMAP embedding of neural retinal and RPE/choroidal cells based on mt-scATACseq data. Cells in **(E-H)** are split by donor and colored based on region of sample.

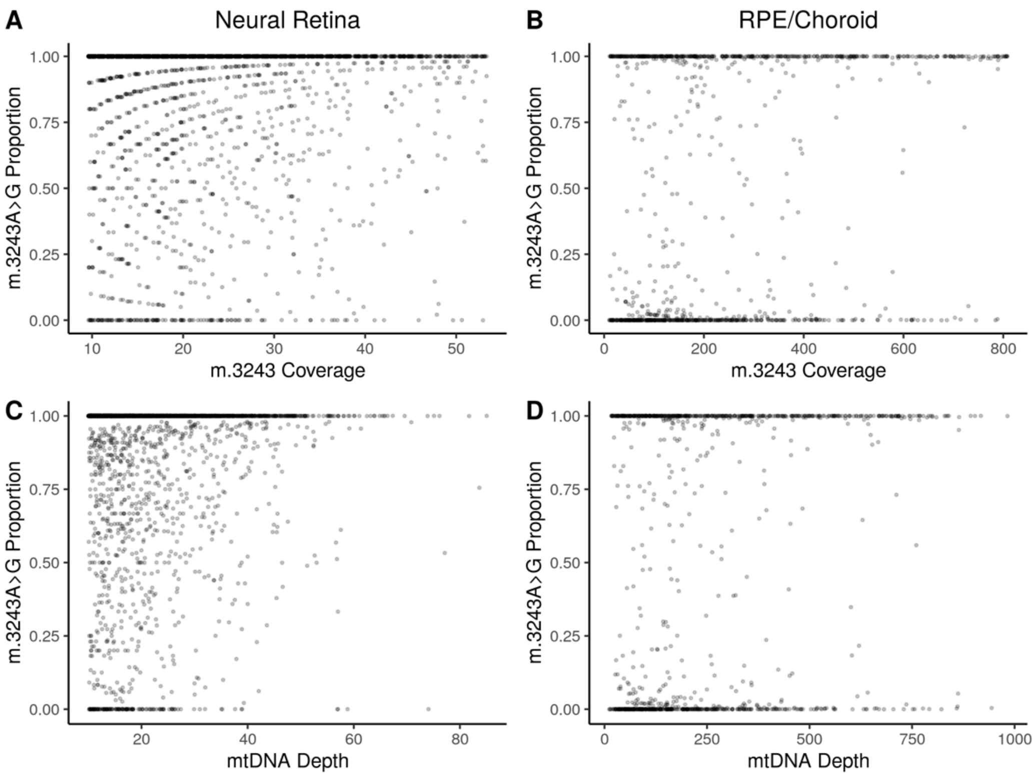

Supplemental Figure 4. m.3243A>G heteroplasmy measurement by mt-scATACseq in the context of locus and genome coverage. (A, B) Each cell from **Figure 3** is plotted with measured m.3243G proportion on the y-axis and mt-scATACseq coverage of the m.3243 locus on the x-axis. No correlation is observed; cells with both high and lower coverage of the m.3243G locus display a range of m.3243G proportions. **(C, D)** Cells from **Figure 3** are shown with m.3243G proportion plotted against average mtDNA sequencing depth. As in **A** and **B**, no correlation is observed, showing that neither sequencing depth nor m.3243 coverage by mt-scATACseq confounds measurement of m.3243A>G heteroplasmy.

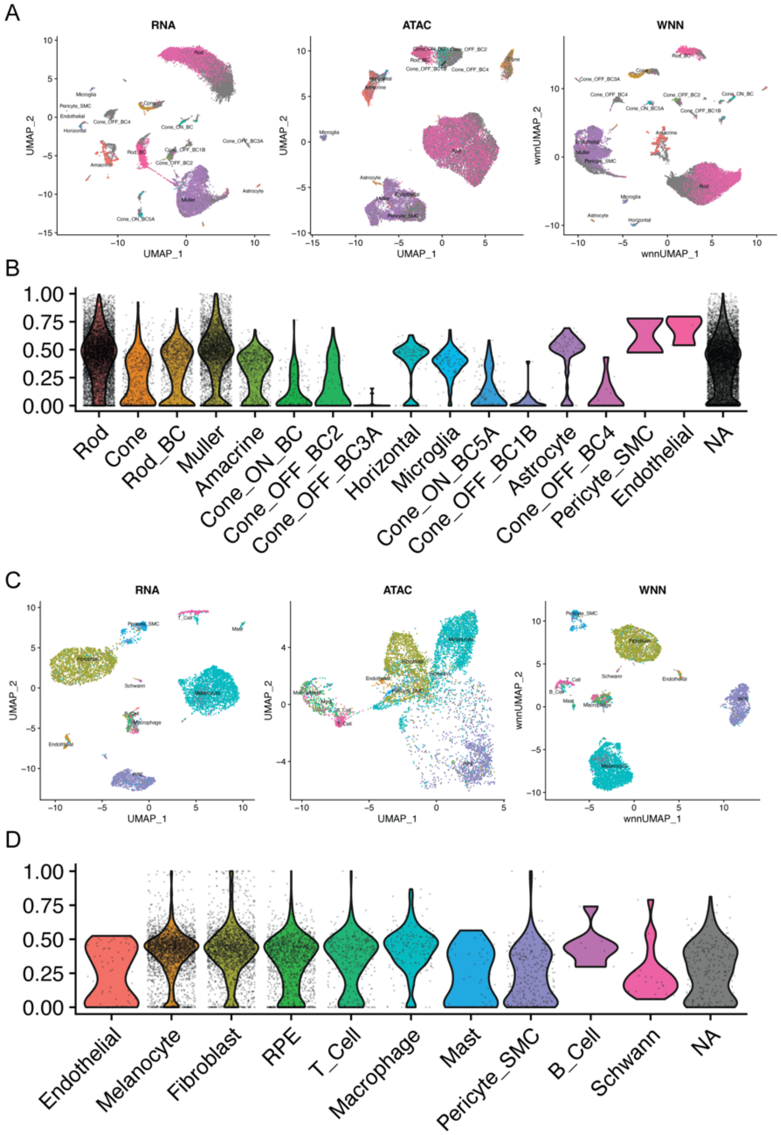

Supplemental Figure 5. Integration of multimodal data from retinal and choroidal single cells. Weighted nearest neighbor (WNN) analysis was used to combine sequencing modalities and drive dimensionality reduction and clustering. **(A)** Two-dimensional UMAP embedding of neural retinal cells based on scRNAseq modality, mt-scATACseq modality, and a weighted combination of scRNAseq and mt-scATACseq modalities. **(B)** Modality weighting in (A) per cell, shown grouped by cell type. **(C)** Two-dimensional UMAP embedding of choroidal and RPE cells based on scRNAseq modality, mt-scATACseq modality, and a weighted combination of scRNAseq and mt-scATACseq modalities. **(D)** Modality weighting in (C) per cell, shown grouped by cell type.
